# Fragmented integration and transnational networks: a case study of Indian immigration to Italy and Spain

**DOI:** 10.1186/s41118-018-0037-7

**Published:** 2018-08-22

**Authors:** Nachatter Singh Garha, Angela Paparusso

**Affiliations:** 1 Centre for Demographic Studies, UAB, Carrer de Can Altayo, Edifici E2, Campus de UAB, Bellaterra, 08193 Barcelona, Spain; 2 Institute for Research on Population and Social Policies (IRPPS-CNR), Rome, Italy

**Keywords:** Indian immigration, Fragmented integration, Diaspora, Transnational networks, Qualitative research, Italy, Spain

## Abstract

According to 2016 municipal register data, Italy has the highest number of Indians in continental Europe (151,000), followed by Spain (41,000). Mass immigration from India to Italy and Spain started in the 1990s, but economic and political environments more conducive to the entry and permanent settlement of immigrants have resulted in more rapid growth of the Indian immigrant community in Italy than Spain. Due to the unskilled and irregular nature of Indian immigration and the lack of integration policies for unskilled labour in both countries, the level of integration of Indian immigrants remains unexplored. In this research, we used a qualitative methodology to explore the integration level of Indian immigrants into different spheres of these host societies. We conducted 86 semi-structured interviews with Indian immigrants in seven cities with high concentration of Indian immigrants in both countries over 2016–2017. We found that the level of integration of Indian immigrants into the host societies is fragmented: some segments of the Indian community are integrated into specific spheres of the host societies, while the rest remain excluded. The main reasons for this fragmented integration are the absence of integration policies for unskilled immigrants, Indians’ provisional attitudes towards permanent settlement in these countries, the internal diversity of the Indian immigrant community and frequent international mobility through transnational networks.

## Introduction

In the first decade of the twenty-first century, Spain and Italy, traditionally emigrant countries, emerged as leading recipients of immigrants from different parts of the world (Arango and Finotelli 2009). According to municipal registers (*Padrón Continuo 2000–2016* in Spain and *Anagrafe 2000–2016* in Italy), the foreign population increased from 1.12 million to 5.03 million (78% non-European Union [EU]) in Italy and 0.92 million to 4.72 million (60% non-EU) in Spain from 2000 to 2016. The share of foreigners (based on citizenship) in the total population also rose from 1.8 to 8.3% in Italy and 2.2 to 10.1% in Spain from 2000 to 2016. This huge influx was driven mostly by the favourable economic environment for unskilled and semi-skilled labour in Spain and Italy after their inclusion in the Euro zone in 1986. This mass immigration has resulted in remarkable population diversity in both countries and posed challenges for local governments, which were quite unprepared for the management of immigrants’ integration into the labour market and society (Einaudi 2007). The major immigrant communities in Italy and Spain are Moroccans, Romanians, Albanians and Chinese.

The Indian community, which is a rapidly growing immigrant group, constitutes only 2.9% and 1.9% of the total foreign population in Italy and Spain, respectively. Despite these smaller numbers, this population has high internal heterogeneity in ethnic origins, skills and socioeconomic statuses (López-Sala 2013; Lum 2010). Despite the rapid growth of Indian immigrants in both countries, researchers have given them little attention in recent years. Studies have mostly described the immigration process (Farjas 2006a, 2006b; Garha et al. 2016a), spatial distribution (Garha and Galeano 2015; Garha et al. 2016b), religion and identity struggles (Garha and Domingo 2017, 2018; Estruch et al. 2007; Denti et al. 2005; Paniker 2007), Punjabi culture, gender relations, family formation (Bonafanti 2015; Bertolani 2017) and Indians’ economic contribution to the dairy industry, agriculture, trade (Sahai and Lum 2013; Bertolani 2005; Beltrán 2004) and hospital services (Gallo 2005, 2008). Very few studies have considered the integration of Indian communities into host societies (Lum 2012; López-Sala 2013).

Italy and Spain were selected as case studies in this research for two reasons: (1) since 2000, both countries have experienced mass immigration from India, with shared sociodemographic characteristics, and (2) both countries are new destinations for the Indian diaspora and have no cultural, political, historical or colonial links with India. It provides us an opportunity to study Indian immigration to new destinations within the Indian diaspora. This paper does not intend to be comparative sensu stricto (data and method do not allow us to pursue this goal). However, we believe that studying the Indian community in two destination countries, such as Italy and Spain, which share some important common characteristics summarised in the so-called ‘Southern European model of immigration’ (e.g. King et al. 2000; Arango and Finotelli 2009)—as far as the management and the policy-making of immigration and integration—is useful in order to shed light on Indian immigrants’ sociodemographic characteristics, patterns of integration, perceptions and opinions of their lives in the country of residence, links with the country of origin, transnational activities and future migration intentions in a common albeit faceted framework, by highlighting similarities and differences between the two countries. This will help researchers to fill an important gap in migration and integration studies, and Italian and Spanish policy-makers to improve their migration (residency rights, regularisations and citizenship) and integration policies (language, training courses, culture and religion) towards an immigrant group, whose size in these two countries and whose international diaspora is of particular interest.

In particular, in this paper, we explore Indian immigrants’ level of integration into different spheres of host societies and the role of their transnational kinship networks in determining the pace and direction of integration. We believe that the diversity of the Indian community significantly affects the pace and direction of the integration process in the host countries. At present, with different origins (Punjab, Haryana, Gujarat, Maharashtra or Kerala), religions (Sikh, Hindu, Muslims or Christians), reasons of migration (manual labour, trade, services or study), education levels (Illiterate, Primary, Secondary or University), occupations (agricultural labour, hospitality workers, salesman and nurses), legal status (irregular or regular) and marital status (single or married), Indians make a very heterogeneous immigrant community in both countries. These internal differences have a significant effect on their pace of integration into the host societies. Additionally, immigrants’ transnational relations, as documented by Garha and Domingo (2017) for the Sikh community in Spain, contribute to provisional attitudes towards permanently staying in host countries and discourage them from investing time and resources in learning the host language, culture and social norms. This, in turn, leads to fragmented integration, which tends to include some segments of the Indian community (e.g. Sindhis and Malayalis) into some specific spheres of the host society (i.e. the labour market) and exclude others.

Our main arguments are as follows. First, Indian immigrants in Spain and Italy live in a provisional state as their ultimate goal is to settle in English-speaking countries (e.g. the USA, the UK and Canada). Their stay in Italy and Spain then is only a step in their whole immigration strategy, and they do not put sufficient efforts and resources into the learning language and customs of the host society. Second, most Indian immigrants are of working age, employed in blue-collar jobs in exchange for very low wages and do not demand anything from the host states’ social welfare departments. Consequently, their host governments feel no threat from them and do not take an interest in their integration.

The paper is structured as follows: The ‘Theoretical background’ section presents theoretical concepts regarding immigrant integration. The ‘Indian immigration in Spain and Italy’ section explains the history and characteristics of Indian immigration to Italy and Spain. In the ‘Data sources and methodology’ section, the data and methods are described, followed by the main results of the analysis in the ‘Results: the level of integration of Indian immigrants into different spheres’ section. In the ‘Integration and transnationalism: quite conflicting processes’ section, we discuss the influence of transnational networks on the level of integration of Indian immigrants, and finally, in the ‘Conclusions’ section, we present some conclusions.

## Theoretical background

The inclusion of immigrant minorities into the mainstream society has been studied through different concepts, e.g. absorption, adaptation, race relations cycle, assimilation, acculturation, inclusion, incorporation and integration (Heckmann 1992: 162–207). The concept of integration is widely used to explain the changing relationship between the newcomers to a residence country and the native or mainstream society. In the recent time, several authors defined integration differently. For instance, Penninx and Garcés-Mascareñas (2016: 14) define integration as ‘the process of becoming an accepted part of society’ and propose ‘three analytically distinct dimensions in which people may (or may not) become an accepted part of society: (i) the legal-political, (ii) the socio-economic, and (iii) the cultural-religious’, while Lucassen (2005) defines integration as a general sociological mechanism that describes the way according to which migrants and non-migrants find their place in a society. It does not produce a unitary and homogeneous society; on the contrary, it allows a number of important differences, which may lead to a multicultural society (ibid.). He further clarifies that the integration of immigrants is an interactive process of learning a new culture, obtaining rights, accessing a new status and building personal relations between migrants and the receiving society. Another crucial aspect of immigrant integration is the degree to which the host society allows the immigrants’ insertion into the host society through its policies, programs and initiatives (Sardinha 2009) and allows them to retain their specific features and identities (Buenfino 2007). Hence, the integration is presented as a two-way process, where immigrants and the host population interact with each other. Moving ahead from this two-way model, Garcés-Mascareñas and Penninx (2016: 2) highlight a major shift in the EU policy framework, starting from 2011 with the renewed European Agenda for the Integration of Third-Country Nationals, which added the countries of origin as a third key actor in the process of immigrants’ integration. Last but not least, during the last decades, integration has been conceptualised as a process (not necessarily straightforward), instead of a goal (Penninx 2004).

**Fig. 1:**
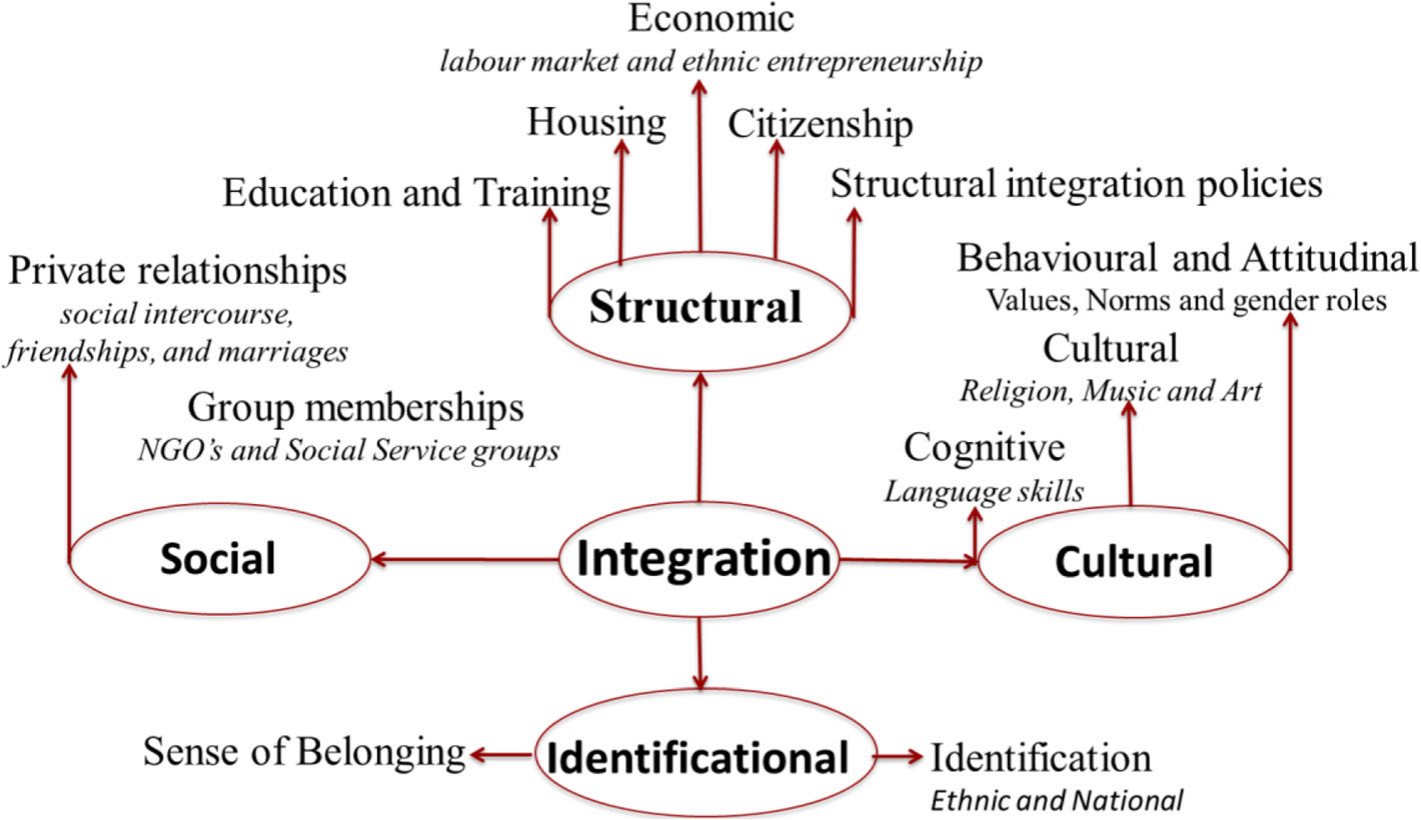
Four dimensions of immigrant integration and related aspects. Source: Heckmann et al. 2001

To measure the integration level, we focus on the four dimensions of integration identified by Heckmann et al. (2001), i.e. social, structural, cultural and identificational (Fig. 1). We have referred to this model for studying Indians’ integration in Italy and Spain, as it is a well-tested model in the European context; it covers all aspects of the process of immigrant integration, and therefore, it provides a complete framework for the analysis of the level of integration of the Indian community in both the countries.

*Social integration* includes participation and membership of immigrants in the private sphere of the host society. It is reflected in people’s private relationships (social intercourse, friendships and marriages) and group memberships (voluntary associations and NGOs). In private relationships, a high rate of interethnic friendships and marriages is generally considered as an indicator for immigrant integration (Alba and Golden 1986; Pagnini and Morgan 1990). *Structural integration* is the acquisition of rights and access to membership of the core institutions of the receiving society, such as education, training, labour market, housing and citizenship. Education is considered as the most crucial factor for the integration of immigrants and their children into the host society (Heckmann 2009). As education is important for the immigrant children, the civic integration is equally important for the adult immigrant population. Recently, most European countries have started civic integration of immigrants. It refers to those policies that push immigrants to learn the local language, civic values and culture of the host country, in order to apply for a permit of stay or the citizenship (Joppke 2007a, 2007b). After education, it is well acknowledged that the standard of housing can affect migrants’ health and quality of life (Lee and Park 2010). Housing also situates migrants in a neighbourhood, a physical and social environment, which provides opportunities to work, access to public services, opportunities to socialise with natives and opportunities to feel more or less secure from crime and discrimination (Phillips 2006). Hence, when measuring migrant integration, the standard of housing, level of segregation, proportion of migrants living in deprived areas and levels of homelessness are often included within the basket of indicators (Ager and Strang 2004; Entzinger and Biezeveld 2003). The acquisition of the citizenship of a host country is considered equally important for the successful integration of immigrants. In the recent public debate, two positions exist on the issuance of the citizenship status to the immigrants residing in European countries (Ersanilli and Koopmans 2011). The first position argues that citizenship is not the end of the integration process, rather it is a part of it. The second position argues that citizenship is the final step of the integration process. Therefore, immigrants holding the citizenship of the residence country are expected to have completed their integration process. These positions can have different implications for the easiness with which immigrants can obtain the citizenship status of a new residence country. Academic literature recognises citizenship status as an objective indicator of immigrant integration (e.g. Diehl and Blohm 2003; Vink 2013), as it helps to reduce gaps between immigrants and natives. However, many migrants show their interest in the host country’s citizenship not only for achieving their political or social rights, but also for gaining autonomy and freedom of circulation, which may facilitate returning to the home country or travelling without constraints towards other countries (Massey et al. 2015). After citizenship, employment is probably the most explored aspect of immigrant integration (Castles et al. 2001). It has consistently been identified as a factor influencing many relevant issues: economic independence, planning for the future, opportunity to develop language skills, restoring self-esteem and encouraging self-reliance (Bloch 1999; Tomlinson and Egan 2002).

*Cultural integration* is a precondition for the participation into the host society, and it refers to the processes of cognitive, cultural, behavioural and attitudinal change. This change concerns primarily the immigrants and their descendants but secondly the receiving society, as integration is an interactive, bidirectional and mutual process. According to Sardinha (2009), cultural integration means embracing different religious beliefs, sexual orientations and cultural affiliations, thus ensuring equal rights for all people living in a society. It is a heterogeneous area, relating to values and beliefs, cultural competences, popular culture and everyday practices. It can be divided into three major spheres: the behavioural and attitudinal sphere (moral attitudes and religious matters), the cultural preferences and practices sphere (music, art and cuisine) and the cognitive sphere (learning language and skills). In the cognitive sphere, language ability is considered fundamental to enhance social mobility and the integration of immigrants into the labour market of the host country (Dustmann and Fabbri 2003). On this regard, recent studies have shed light on the positive relationship between socioeconomic integration and language proficiency (e.g. Di Bartolomeo and Strozza 2014). Age of immigration, length of stay, parents’ background and higher education level facilitate the cultural integration of immigrants (Alba and Nee 1997) and in particular the acquisition of the host-country language (Esser 2006; Luciak 2004). Finally, *identificational integration* refers to subjective sense of belonging and identification, particularly in forms of ethnic and/or national, regional or local identifications (Heckmann 2007). Sense of belonging to the host country is extremely important for the integration process of any immigrant group, since it strengthens the attachment to the host society and reinforces the intention of a permanent and durable residence abroad (Barbiano di Belgiojoso 2016). The personal identity of immigrants and its recognition in a host society is also relevant in the process of integration (Heckmann 2007). It is also politically relevant whether the immigrants identify themselves with other immigrants in efforts to gain recognition and rights or share a collective social identity with the host population (Westin 2003). The four dimensions of integration will be disentangled in the ‘Results: the level of integration of Indian immigrants into different spheres’ section and analysed with respect to Indian immigrants.

In this paper, the concept of *fragmented integration* refers to a situation when some segments of an immigrant community are integrated into specific spheres of the host societies (mainly economic), while the rest remain excluded. It commonly happens with the immigrant groups from countries with high ethno-religious and socioeconomic diversity, like India. Immigrants coming from different backgrounds integrate at a different pace, but due to our tendency to treat all immigrants from a country alike (so-called methodological nationalism (Faist 2012)), we undermine these differences and we tend to consider all immigrants as a single homogeneous unit.

## Indian immigration in Spain and Italy

Pioneer Indians entered Italy as British soldiers during the Second World War, but they returned after the war (Bedi 2011). In the 1960s, an immigration flow composed of theology students (in Catholic churches and convents), priests, nurses and housemaids from Kerala (a south Indian state) and central parts of India entered and settled permanently in the capital region of Rome and northern parts of Italy (Gallo 2005; Sahai and Lum 2013). On the other side, pioneer Indian immigrants in Spain settled in the Canary Islands in the 1950s (Navarro 1974). They were merchants from the Sindh province of undivided India and came with the sole motive of trade (López Sala and Sánchez 2010). Their number was limited to a few hundred and they had limited contact with the host society (López-Sala 2013). These first movements of Indian unskilled workers, students and traders towards Southern European countries marked the routes of immigration for further immigration.

**Fig. 2:**
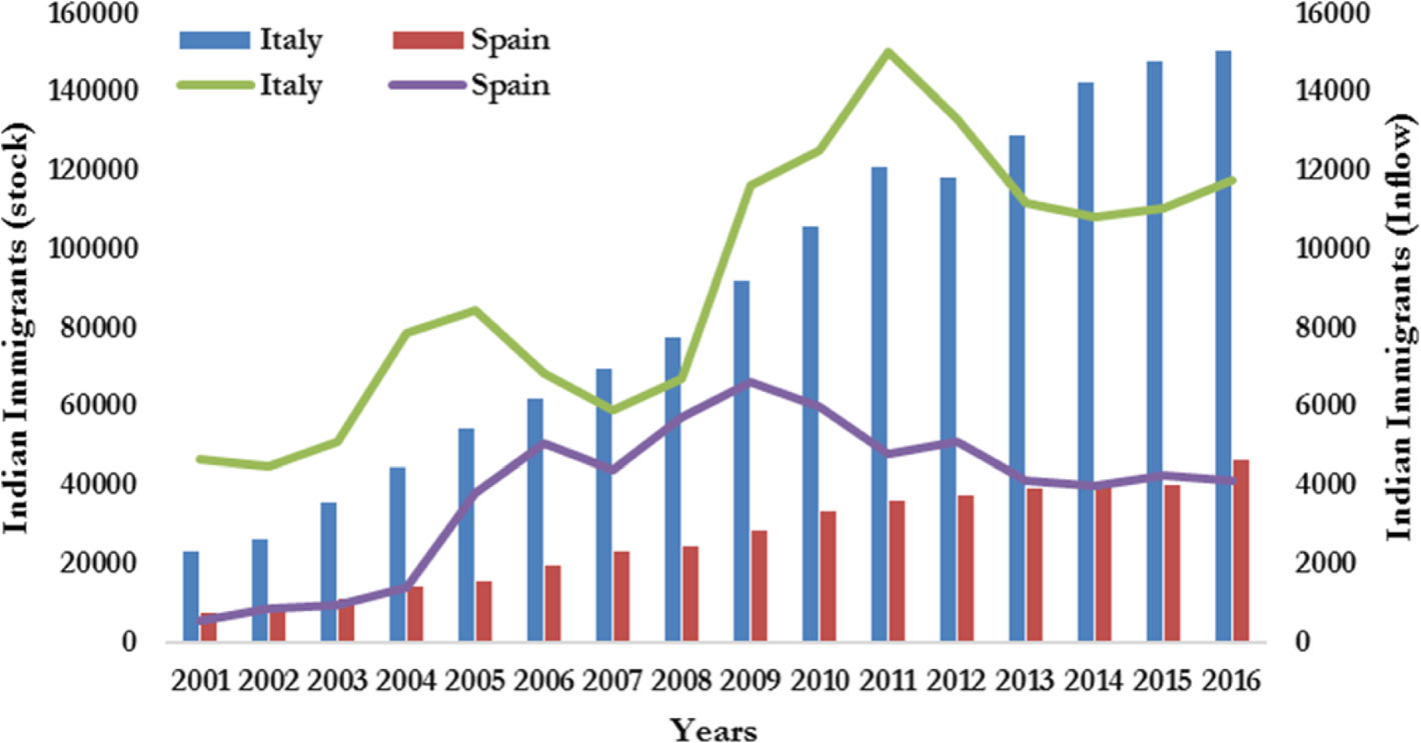
Annual flow and stock of Indian immigrants to Italy and Spain, 2000–2015. Source: own elaboration, from municipal register, 2000–2015, INE, Spain, and ISTAT, Italy

Following the neoliberal turn in India’s economic and foreign policy, mass immigration from India to Italy and Spain started in the 1990s (Garha et al. 2016b). This immigration was featured by the male unskilled labour force coming from the northern Indian states of Punjab and Haryana (Garha and Domingo 2017). It coincided with the immigration boom in both countries (Peixoto et al. 2012). In Italy, the 1990s immigration law (Law 39/90, so-called Martelli Law) facilitated the permanent settlement of immigrants (Einaudi 2007) and opened Italy for unskilled labour immigration (Bonifazi 2007). Similarly, in Spain, the First Immigration Law of 1985 (*Ley de Extranjeria 07/1985*) with all its amendments paved the way for permanent settlement of immigrants (Aja 2006; Moya 2006), including Indians. After the first settlers, transnational kinship networks played a major role in attracting new immigrants from India and other diaspora locations (Garha and Domingo 2017). From 2000, the flow and stock of Indian immigrants multiplied several times, till the economic crisis engulfed both countries and slowed the inflow (Fig. 2).

According to ISTAT, the annual inflow of Indian immigrants to Italy doubled in the last 16 years, passing from 4.6 thousand annual immigrants in the year 2000 to 11.6 thousand in the year 2015 (Fig. 2). This inflow has witnessed a significant increase in the period of 2002–2004, which was partly due to the direct migration from India and partly related to the regularisation process of irregular immigrants who were already present in Italy. However, this sharp increase was followed by a steep decline till 2006. On the contrary, from 2006 to 2010, despite the economic crisis, the number of Indian immigrants increased three times with a high annual growth rate. In 2010, the inflow reached its peak with 15 thousand immigrants entered only that year. After 2010, it started declining again and now it is around 11 thousand immigrants per year. The most important characteristics of that inflow are its young age structure and the skewed gender composition in favour of males. As in North India, males are still considered as breadwinners, and they mostly migrate to earn livelihood and to settle in other countries. Consequently, initial inflow was mainly composed of young males, although the proportion of females started increasing when males started bringing their families from India to Italy.

This regular inflow of Indian immigrants has contributed to the formation of the sixth largest foreign community in Italy. According to ISTAT, in the year 2000, only 22 thousand registered Indian immigrants were living in Italy, but their number increased rapidly to 121 thousand in the year 2010. After 2010, under the impact of economic crisis and the shortage of the labour opportunities, many Indians left Italy and moved to other countries, some of them also returned to India. Consequently, despite of the highest inflow in 2010, the size of the Indian community reduced to 118 thousand individuals in the year 2011. After this small decline, the size of the Indian community again started to grow rapidly in the coming years and reached its peak, for instance 150 thousand in the year 2015 (see Fig. 2). The gender composition and the age structure of the stock of the Indian immigrant population is characterised by male dominance and concentration in the young age groups.

In Spain, according to INE, the annul inflow of Indian immigrants in the year 2000 was merely around 539 individuals, but this number increased sharply to 6.6 thousand in the year 2008. It was mainly due to the regularisation program of 2001 and 2005 that attracted a large number of Indians, who migrated directly from India or entered the country from the neighbouring countries like France, Germany or Nordic countries, where they were living irregularly. Later on, owing to the negative effects of economic crisis, this flow declined, amounting to around 4 thousand individuals per annum. As a result of this inflow, the total population of Indians in Spain increased from 7 thousand individuals in the year 2000 to 46 thousand in 2015. Like in Italy, the Indian community in Spain is male dominated and mainly concentrated in the working age group of 15–49 years.

To sum up, we can argue that the history of Indian immigration in Italy and Spain fits the so-called Southern European model of immigration. In particular, the main characteristics of this model are (a) the timing and the size of inflows, (b) the reasons for and the modes of entry (a lack of selective immigration policies and the use of ex post instruments to provide a legal status to immigrants, such as regularisations, quota systems and flow decrees) and (c) the distinctive way of integration into the local labour market (a large underground economy attracting undocumented immigrants and a strong segmentation of the labour market) (Peixoto et al. 2012; Di Bartolomeo et al. 2016).

## Data sources and methodology

The data has been collected through 86 semi-structured interviews conducted by the first author, during the period between January 2016 and June 2017. Interviews were conducted in seven cities where Indians are mainly concentrated in Italy (i.e. Rome (13,702 individuals listed in municipal register 2016), Brescia (15,028) and Latina (10,003)) and Spain (Barcelona (5895), Valencia (2276), Madrid (2262) and Santa Cruz de Tenerife (1146)). Interviewees were selected through snowball sampling technique (Johnson 2014). All socioeconomic and demographic characteristics of participants, like age, gender, place of residence, education, legal and marital status, year of arrival, employment and religion, were taken into consideration (Table 1). The interviews were conducted with a semi-structured open-ended questionnaire, and respondents were asked to express themselves on the following issues: their immigration history, transnational networks, family background, education and training, religious affiliations, entry into labour market, access to public institutes in the host country, regularisation process and citizenship, marriage and family reunion, participation in social sphere (friends, partners or social groups), awareness of local politics, children’s education, neighbourhood relations and future expectations. Average time of interviews was 60 min, and interviews were conducted in one of the three languages, i.e. Hindi, Punjabi or English, as per the convenience of interviewees. Most of the interviews were conducted at the interviewees’ usual place of residence or at some public places selected by them. All interviews were audio recorded.

**Table 1:** Sociodemographic characteristics of the interviewees in Italy and Spain

Sociodemographic characteristics	Interviewees	
	Italy	Spain
Sex		
Male	26	34
Female	12	14
Age		
Young adults (16–30 years)	12	16
Adults (31 years and above)	26	32
Religion		
Sikh	22	30
Hindu	12	16
Christian	4	0
Muslim	0	2
Education		
Primary	18	26
Secondary	14	14
University	6	8
Occupation		
Agriculture	11	9
Factory workers	6	7
Services	11	15
Unemployed	7	13
Students	3	4
Duration of stay		
1 year	3	8
1 to 5 years	14	16
5 and more years	21	24
Marital status		
Never married	15	16
Married	22	30
Others	1	2
Legal Status		
Irregular	4	6
Regular	28	34
Naturalised citizen	6	8
Generation		
First	32	36
One and half	6	12
Total	38	48

Source: compiled by the first author on the basis of in-depth interviews in Spain and Italy during 2016–2017

We have used qualitative research methodology. The inductive approach, also called a bottom-up approach, under ‘grounded theory’ (Glaser and Strauss 1967) has been applied. All interviews were encoded on the computer programme Atlas.ti, using a thematic classification offered by Boyatzis (1998). After transcribing the interviews, following the steps mentioned by Braun and Clarke (2006) for thematic analysis, we searched for the issues highlighted by the interviewees in their discourse and coded the content of interviews with initial codes. We formed families of primary codes to classify the information related to one theme in one place from all interviews and prepare the primary data for the analysis. Then, we searched for the patterns and themes repeated in all interviews. We selected quotes to present different views regarding the themes under study. Finally, we prepared a report on the overall pattern and trends regarding the integration of Indians into different spheres of the host societies.

## Results: the level of integration of Indian immigrants into different spheres

### Social integration

Social integration refers to people’s ‘private relationships’ and their ‘associational membership’ in the host society. In private relationships, we include both interethnic friendships and mixed marriages. Among Indians, private relationships are mainly limited to their own ethnic and religious groups. Despite their long stay in both countries, most of them have very weak social ties with natives, which show their low integration to the host societies. Generally, they point out existing cultural differences with the host society as a prime reason for this. Amandeep, 23, female, a restaurant worker from Barcelona states that ‘I don’t have friends from the host community. Our cultural differences like language, eating habits, socializing and way of thinking, make any interaction very difficult’.

As already elucidated, most of the Indians are economic migrants. They are doing labour-intensive jobs, like agriculture and services (in particular, sales, restaurants and hospital services), where they mostly work with their countrymen or other immigrants. Therefore, they get very few opportunities to make friends and socialise with host population. Subhash, 45, male, a salesman in Madrid, says ‘my all friends have come from India and are immigrants like me. We work and live together. We don’t have time to make local friends’. Sometimes, the seasonal nature of their work and long working hours make it difficult to get connected with the local community. Parminder, 28, male, an agricultural worker in Latina, explains ‘I work from 6 am to 6 pm. After finishing my work, I return directly to my apartment. Because of seasonal work I keep on moving from one farm to another. I have least contact with the host community’. Another main problem is less command over host languages. A common language provides a bridge of communication for any human interaction and encourages interethnic friendships; contrarily, the lack of language skills creates a barrier for any possible interaction. As explained by Mohani, 32, female, a housewife in Valencia, ‘our main problem is language. Mostly Indians do not learn Spanish before immigration …. Without common language we fail to communicate with local people. It cuts our chances to have local friends’.

Recently, with the occupational diversification among Indians, many of them are coming forward to have relations with the host community and other immigrant groups, like Kailash, 36, male, a travel agent in Barcelona, explains ‘by changing my occupation from an agricultural worker to a travel agent, I have widened my friends circle. Apart from other immigrants, now I have friends from the host community also’. The situation is much better for the younger generations, who have studied in the local schools, like Gagan, 22, female, a student from Barcelona, says ‘In school I come in contact with many students from different nationalities, now I have friends from Pakistan, Ecuador, Bangladesh, Peru and some local girls also’, but their number is very small.

Among Indians, mixed marriages are very rare, mainly because of religious and culture differences with the host societies. In the Indian community, religion plays an important role in marriage ceremonies. Hence, the restrictions imposed on the inter-religion marriages among traditional Sikhs and Hindus affect the number of mix marriages; as explained by Prakash, 44, male, a Hindu in Valencia, ‘in our Hindu marriage system bride and groom, should be from the same caste and religion. Otherwise, they are not allowed to marry’. Most of the young men and women in both countries also want to marry in their own community in India, as they feel that in the host societies the institution of marriage has lost its importance, like Sandeep, 23, female, a computer professional in Barcelona, says ‘I will marry in India. Here in European society marriage has lost its importance. People live together without marriage, and if they marry they divorce in few months’. Sometimes, the differences regarding the gender roles and the division of work at home observed during the cohabitation with natives also discourage young adult males to marry in the host communities, as explained by Inderjit, 24, male, restaurant worker in Barcelona, ‘I have a girlfriend here [in Barcelona], but I want to marry in India [with an Indian girl]. Especially after spending some time with her, I felt that I should marry in India, in our own culture, where gender roles are clearer’. Mixed marriages as an indicator of integration is also questionable because sometimes these are used instrumentally by migrants, as a source of residency or citizenship permits, like Avtar, 47, male, an unemployed in Barcelona, admits that ‘I have done a paper marriage with a Spanish woman, to regulate my legal situation in Spain. I have paid her 6000 euros for this’. Interethnic friendships and marriages between Indian immigrants and natives are rare in both the analysed countries; this feature confirms their low integration to the personal sphere of the host societies.

As per the associational membership is concerned, Indians have very low participation in the host sociocultural or labour associations. Lack of information regarding immigrant NGOs is the main reason behind their low participation. Several religious places (like Gurudwaras ‘Sikh temple’ and Hindu temples) provide them platforms to socialise within their own group, but it limits their interaction with the host societies. Majority of Hindus in Spain and Italy associate themselves with the Hare Krishna Group, which has temples in Barcelona, Madrid, Valencia and Rome. On the other side, the Sikh community has organised itself around local Gurudwaras, which are the centres of their social life (Sikh temples, 22 in Spain, see Garha and Domingo 2018, and 62 in Italy). As explained by Baljeet, 35, female, a housewife living in Rome, ‘we have a Gurudwara [Sikh Temple] in Rome, where we spend all our free time with friends and other community members, we hardly go out for socializing’. Finally, although most Indians belong to the working class, their participation in labour unions is also limited to some parts of the Brescia region in Italy. Sindhi business community in some cities of Spain, like Barcelona and Santa Cruz de Tenerife, has organised itself in some *Industani clubs*, but they are also limited to business elites and they have very limited contacts with the host society. In sum, we can conclude that in both countries, immigrants’ social participation is quite low, although, contrarily to Spain, some Indian workers have started to participate in worker unions’ activities in Italy.

### Structural integration

In the Indian community, the education of kids is considered as a very important issue. The primary education is free of cost and available to all children in both countries. Mostly Indian parents enrol their children in public schools, but a majority of them are unsatisfied with the standard of education. On this regard Daljeet, 35, a housewife from Brescia, says ‘I have enrolled my children in a public school. Here [in Italy] the access to public schools is easy, but the quality of education is lower as compared to schools in India. Especially, they don’t teach English. Private schools are good, but they are very expensive, which we can’t afford’. Secondly, most Indian parents are also worried about the segregation of immigrant students in public schools and neglect of public education system on the part of local governments. According to interviewees, Indians who afford the fees also send their children to private schools. For example, Gurnaib, 34, male, a restaurant owner in Valencia, says ‘In public schools you will find only immigrant children, upper-middle class Spanish families send their children to private schools … that is why nobody cares about public schools. I also enrolled my children in Cambridge school’. Owing to the lack of trust in the public education system, some parents encourage their children to enrol in professional courses, in order to have more chances to get a stable job in the future, like Avtar, 46, male, a factory worker in Brescia, says ‘I encourage my sons to enrol in a professional course, here [in Italy] the higher studies are difficult and most of the immigrant students don’t receive any job after completing their degrees. It is better if they learn some professional traits, so in future at least they can earn their livelihood’. Some Indian students have also noticed discrimination regarding the availability of scholarships to immigrant students who want to continue their higher studies, as explained by Ramandeep, 28, male, a student in Rome, ‘There is no discrimination with immigrant students in primary schools, but when it comes to the higher studies, there exists some discrimination with them. School management encourage them to opt for professional courses, and often they do not receive any scholarship for higher studies’. To sum up, we can say that the public education system in both countries is not fulfilling the aspiration of Indian parents regarding their children’s education. Consequently, most of them want to move on to an English-speaking country, where they think that their children will get better education. The stated discrimination in the upper education in Italy is a matter of concern also for the Indian community, as it reduces the chances of immigrant students to have higher education, thus affecting their upward social mobility in the host society. Conversely, in Spain, we have not registered any kind of discrimination in the public education system.

As argued in the ‘Theoretical background’ section, many European countries have recently implemented civic integration policies, with the aim of enhancing and, to some extent, controlling the level of cultural integration of adult immigrants in receiving societies (Paparusso 2016). According to these policies, the immigrant’s success in civic integration courses implies the issuance or the renewal of a permit of stay and the granting of the citizenship status. In Italy and Spain, municipal council entities provide civic and language courses for the immigrant population. In Spain, these courses are freely available for all immigrants including irregular immigrants, whereas in Italy, owing to the limited resources and infrastructure facilities, these courses are not fully operational yet and only available for regular immigrants (Paparusso 2016). In Spain, the basic knowledge of Spanish and another regional language is a must for all applicants of continuous regularisation process (*Arraigo*^1^). Therefore, all irregular Indian immigrants apply for these language courses. However, many of them consider these courses as insufficient for proper learning of the host languages, but they accept that these courses help them to regularise their legal status in the country, like Harwinder, 34, male, an irregular immigrant in Barcelona, explains ‘I have done two language courses because now it is compulsory for the papers [regularization]. I still don’t speak Spanish or Catalan properly, but I have school certificates to fulfil the requirement of Arriago’. On the other side, in Italy, the knowledge of the Italian language (level A2) and culture is needed to renew the permit of stay, to apply for the long-term residence permit and indirectly to apply for naturalisation. But opposite to Spain, in Italy, all the language courses are reserved for regular immigrants. Hence, in a significant number of Indian immigrants who have entered irregularly in Italy or become irregular after arrival, the possibilities to learn the Italian language are extremely low. As explained by Param, 26, male an irregular immigrant in Rome, ‘In Italy if you don’t have papers, you cannot enrol in any public school, it makes very difficult to learn host language and get connected with the local community’.

Moreover, in both countries, employment offices, immigrant, catholic and no-profit organisations set up various professional courses for unskilled immigrant workers. However, the participation of Indian immigrants in the professional courses is still quite limited. It is mainly owing to the lack of information about these programs and the limited number of seats available in different courses. As explained by Kuldeep, 30, male, an unemployed in Barcelona, ‘I have heard that the ‘INEM’ [employment office] offers some professional courses, but the information does not reach us on time. Every time when I go there they have no vacant seats’.

Indian immigrants in both countries have a high degree of residential segregation in poor housing areas (see Garha et al. 2016a for Indians in Spain). Low rents, easy availability of apartments and their will to settle close to their relatives and religious places are the main reasons behind this residential segregation (Garha and Galeano 2015). According to Surjit, 54, male, a shopkeeper in Barcelona, ‘In Barcelona good apartments are very expensive, that is why most of the Indian immigrants are living in deteriorated residential buildings of El Raval, Badalona or Hospitalet de Llobregat, which is the cheapest option in housing market’. In Spain, most of the Indian immigrants face economic exclusion from the good residential areas, but there are no recorded cases of discrimination against immigrants in the housing market. On the contrary, in Italy, some of the interviewees highlighted the discrimination in the housing market, which indirectly forces the immigrants to settle in poor residential areas, like Mandeep, 31, male, factory worker in Brescia, says ‘many Italians do not rent their apartments to immigrants, especially, Asians or Africans’. This economic exclusion or discrimination based on origin contributes to the segregation of Indians in poor immigrant neighbourhoods, where they spend most of their free time with their countrymen or other immigrants, which reduces their chances to get integrated into the host society. Like Karamjit, 32, male, an agricultural worker in Latina, states that ‘I live in an immigrant neighbourhood, where apartments are deteriorated, but cheap. No Italian lives in this area, so I hardly meet anyone here’.

As theorised in the so-called Southern European model of immigration (e.g. King et al. 2000; Arango and Finotelli 2009), irregular immigrants largely contribute to the Italian and Spanish informal sector of the labour market, particularly at the lower skills level. It results in the definition of immigrants’ residence rights primarily in economic terms and as temporary workers. As most of the Indian immigrants enter irregularly in both countries or become irregular after arrival and depend on the immigration laws of the host countries to regularise their legal status, the regularisation processes in both countries play an important role in affecting their status mobility and level of integration. Since 2000, in Spain, the *Arriago law* has been initiated to control the number of irregular immigrants in the country. Although it is a lengthy process and has some serious weaknesses, it has helped 5745 irregular Indians to regulate their legal status during 2009 to 2015, who have entered irregularly in Spain and were living there for more than 3 years. According to Jasbir, 41, male, a chef in Valencia, who get regularised through *Arraigo*, ‘The main problem with the ‘Arraigo’ law is its necessary pre requisites, as it requires three years of registered uninterrupted stay in Spain (without work and residence permission), knowledge of host languages, a full time work contract, police clearance certificate from the country of origin and a valid passport. Many Indians fail to fulfil these requirements’.

Unlike Spain, Italy does not follow a continuous process of regularisation of irregular immigrants. However, starting from the late 1970s, periodical amnesties have been used by the Italian governments as an ex post instrument to allow the regularisation of many immigrants in Italy and therefore the granting of residency rights (Paparusso et al. 2017). The uncertainty regarding the next amnesty negatively affects the settlement intentions of irregular immigrants that hinder their pace of integration. As explained by Hardeep, 33, male, a factory worker in Brescia, ‘here [in Italy] you don’t know when the government will announce next amnesty for irregular immigrants. This insecurity declines the stability of immigrant population and they always keep looking towards other countries, like Spain or Portugal for regularization’. In Spain, the *Arraigo* law gives hope to the irregular immigrants that if they stay in country, learn the language and arrange a work contract, after 3 years, they can become a regular resident. It works as a positive trap for irregular immigrants, because it pushes them towards integration. Conversely, in Italy, the uncertainty regarding the regularisation contributes to the provisional attitude of immigrants and do not provide them space for integration.

Generally, regularisations in both countries issue a temporary residence and work permit for 1 year, and its renewal depends upon the tax paid during the period of stay, the availability of a regular work contract, a minimum income and proper housing conditions. Immigrants who fail to fulfil these requirements may lose their residence permit and become irregular again. Among the interviewees, this does not occur very frequently: four in Italy and two in Spain have lost their initial residency permit, due to the lack of work contract or less tax paid in the first year of stay. This supervening irregularity among Indian immigrants is more visible in Italy than Spain. As according to Satnam, 55, male, a salesman in Rome, ‘a significant part of irregular immigrants of Indian origin in Italy entered with working visas of six month or nine months, which they often fail to renew and become irregular. Also sometimes people provide some fake work contract to get the working visas for their relatives and when they reach Italy, they don’t find any work contract to renew their permits’. We know that moving from a regular to an irregular status has a very negative impact on immigrants’ lives and on their status mobility in the country of residence, because of the difficulties to re-fulfil requirements (Paparusso et al. 2017).

In Spain and Italy, the acquisition of citizenship is a lengthy process, which for non-EU migrants, like Indians, takes 10 years of uninterrupted legal stay in the country and some knowledge of the host language, history and culture. The new criterion based on civic exams in Spain has declined the chances of many Indians to become a naturalised citizen of their country of residence, like Resham, 35, male, a restaurant worker in Barcelona, claims that ‘I want Spanish citizenship, but now they have placed an exam also, I don’t know if I will get it or not’. In Italy, no citizenship test is required for naturalisation; however, the knowledge of the Italian language and culture is required for the immigrants who apply for host citizenship in a discretional way, as elucidated before. Thus, the limited knowledge of the Italian language can represent a problem for those Indians who want to acquire the Italian nationality. In Spain, Latin American immigrants need only 2 years of regular residence to apply for the citizenship status. This discrimination hurts sentiments of other immigrants, as expressed by Jagtar, 34, male, a salesman in Madrid, ‘this positive discrimination with Latinos, makes us feel as second-class citizens. It is against the principles of equality before law and shows the double standards of the Spanish government’. Deprived of the host citizenship, most Indians feel less attachment or sense of belonging with the host countries, which results in their low integration into the host society.

In particular, most of the Indian immigrants consider citizenship as a key that can open the door of the Western world, for them and for their children. On this regard, Satnam, 55, male, a salesman in Rome, says ‘I think the citizenship is must for everybody, especially for children. With European citizenship they can move to any country’. Hence, for the Indian immigrants, it is difficult to conclude that the host citizenship positively or negatively affects their integration process. Moreover, Indians in both countries declare their intention to move to the UK. Although our respondents do not directly mention Brexit in their narratives, we believe that it may affect their long-term migration intentions, changing their preferences for other English countries. For instance, we could make the hypothesis that the fact that some of them have started thinking to migrate also to Canada, where they have some kinship networks and think their children will have a better future, could be partly related to it. However, our interviews do not allow us to verify this hypothesis.

Despite their low socioeconomic profiles, Indians have been significantly contributing to the labour market and economic development of the host countries, especially by filling the jobs, like agricultural labour, industrial worker or hospitality services, which the native population are reluctant to do (Sahai and Lum 2013). Nevertheless, Indian immigrants are mostly trapped in the low-paid blue-collar jobs, which affect their upward economic mobility and integration into the host society (Lum 2010). The host communities generally appreciate them because of their honesty, limited demands, easy availability and sense of responsibility. Manjeet, 40, male, an agricultural worker from Brescia, says ‘Indian immigrants in Italy are mostly engaged in manual jobs, like cleaning of cow sheds and agricultural work, which the local people don’t want to do … local farmers prefer us because we do these jobs at very less price, without demanding worker’s rights’. As a result of their occupational segregation in low skilled jobs, they have very limited contact with the host working class. It reduces their social circle to their countrymen or other immigrants, with whom they share their work places.

Owing to the recent economic crisis (2008–2014), most of the Indian parents feel insecure about the future of their children in both countries. The high unemployment rate, even for the native population, is their major concern. Malu, 48, female, a shopkeeper in Santa Cruz de Tenerife, explains that ‘My son has completed a diploma here [in Spain], but there are no jobs in Spain, even the local people are immigrating to other countries for work, may be in future my son will also migrate for work’. Even in the Sindhi community, which is an economically well-established business community, economic uncertainty is increasing. They are also worried about the continuation of their family businesses, and some even have diverted their attention towards services and public administrative jobs, which can be seen as a positive move towards integration. Chandru, 53, male, a shopkeeper in Santa Cruz de Tenerife, Spain, shows his concern as ‘Businesses are not going well. I think our next generations will have to leave our traditional occupational niches and look for jobs in other sectors’.

Another important aspect is the limited participation of females in the host labour market. In the Sikh community, which makes the majority in both countries, the participation of females in the labour market is considerably lower than Malayali (who mostly work as nurses) and Sindhi females (who are mainly engaged in family businesses). This low participation in the labour market reduces their contact with the host society and adversely affects their integration, as in the words of Vasundra, 34, female, a nurse from Rome, ‘here in Indian community women are mostly limited to their houses. They are unemployed and do not go out in search of work. It reduces their contact with Italians and affects their level of integration into the host labour market’. To sum up, we can argue that Indian immigrants, especially males, in both the countries have higher employment rates, compared to other immigrant groups, but they mostly work in the unskilled sector of the host labour market; this affects their upward economic mobility and integration into the host society.

### Cultural integration

In the behavioural and attitudinal sphere, we include the moral attitude towards gender roles, divorce, cohabitation and homosexual relationships. In the Indian community, there is a clear divide regarding the perceptions of the host society. There are some Indians, like Gurwinder, 24, female, a factory worker from Valencia, who claims that ‘the host society is an egalitarian and free society, where all individuals have full freedom of choice, without any racial and gender discrimination’ and admires the openness of the host culture. However, for the majority of Indians, the host society is excessively open, individualist and lacking family values, like Swaran, 48, male, a shopkeeper in Barcelona, says ‘here [in Spain] the society is excessively open, young boys and girls wear short cloths, call their parents with their names, and drink or smoke in front of them, which is unacceptable’. Similarly, regarding divorce and cohabitation, the majority of Indians have traditional views, like Jayant, 49, male, a wholesaler in Valencia, says ‘European society is a mess, here young boys and girls start cohabiting without marriage … they change their partners like dresses and don’t want kids … mostly they don’t marry, but if they do, they get divorced soon’. Owing to their religious beliefs, most of the Indians are against same-sex marriages. As explained by Kulwant, 37, male, a milkman in Brescia, ‘In our culture it is clear that each man for a woman, and each woman for a man, but here [Italy] boys are marrying with boys and girls with girls … there is no sense at all’. When it comes to gender roles, in the Indian community, the division of work is also very traditional: males are breadwinners and females are housewives. There are some men, who do not allow their wives to go out for work, but most of the women claim that their husband has no problem with their desire to work. Mostly they do not get any job due to the responsibility of kids and the lack of host language skills. Sarabjeet, 34, a housewife in Rome, says ‘My husband goes out for work and I manage home and children. I want to work outside, and my husband has no problem with it, but now I have two kids and I want to give time to my kids’.

Religious matters include religious practices, like attendance of places of worship, food avoidance for religious reasons, attitudes towards both host and origin country festivals and attitudes towards being a member of a religious association. Among the Indian immigrants in both countries, majority belongs to the Sikh religion, which is broadly divided into Moderate (90%) and Baptised Sikhs (10%) (these estimates are provided by the Gurudwara managing committee heads in both countries), followed by a small number of Hindus, Christians and Muslims. Owing to their unique physical appearance (with turban and long beard), outfits, diet (purely vegetarian) and lifestyle (no smoking and no drinking alcohol), baptised Sikhs find it difficult to fit in the host culture. Gursharan, 24, male, a baptised Sikh from Barcelona, argues that ‘I cannot fully adopt Spanish culture, because I have everything different, starting from my physical appearance, my cloths, my food, my music, my way of living, everything is different’. It is also a big problem for the school-going baptised Sikh children, who got bullied by their classmates and cannot share meals with other kids. Kuljit, 37, female, mother of two baptised kids in Brescia, explains that ‘Here [in Brescia] in schools, children tease my sons for having long hairs. It puts enormous stress on their young minds. Secondly, my kids are vegetarians and in school they serve meat in the midday meals, so I have to bring them back to home for lunch and it reduces my chances to work outside’. Baptised Sikhs also face bans on wearing their religious symbols at public places, which includes a sword *Kirpan*. Sukhchan, 42, male, a Gurudwara committee member in Madrid, explains that ‘as a baptised Sikh I am not allowed to undress my sword, but here when police caught me with sword, they snatch it and fine me … now because of this ban I don’t go out of Gurudwara sahib with sword’. They also face discrimination in the labour market as many employers (especially in the hospitality and sales sector) hesitate to hire them because of their different look. Some of them even change their physical appearance to be eligible for jobs in the hospitality sector. Ravinder, 26, male, a restaurant worker from Valencia, who changed his look, explains that ‘I was a baptised Sikh in India, when I reached Spain my friends told me that if you don’t trim your hairs, you will not find any job in Spain, so I trimmed my hair and now I am working in a restaurant’. The number of baptised Sikhs is very small as compared to the moderates, who have adapted their lives according to the cultural norms of the host societies. Majority of them have cut their hairs and modified their eating habits to get mixed in the host society and get jobs in restaurants and hospitality sector.

Another main problem is related to the opening of new Gurudwaras or Temples in the host countries. After the religion-based attacks in many European cities, people feel unhappy with the opening of any new religious places in their neighbourhoods. In Spain, the local population stopped the construction of Gurudwara in various parts of Barcelona, as explained by Karan, 36, male, a baptised Sikh in Barcelona, ‘Local people were against the inauguration of Gurudwaras in Salt, Olot and Santa Coloma de Gramanet municipalities. They confuse us with Muslims, and saw it like an encroachment of their space by a foreign religion’. It has damaged the relationship between the Sikh community and the hosts in many cities. In comparison to the Sikhs, the Indian Christians and Hindus do not have such problems with the host society. Indian Christians settled in Italy feel blessed to be in a Christian country. Among the interviewees, most of the Indian Hindus have also expressed their freedom to practice their religion in the host countries, like Prem, 54, a Hindu priest in Krishna Temple in Barcelona, says ‘here [in Spain] people are very kind to all other religions of the world. They give full respect and freedom to practice any religion. Even there are many Catalans, who have converted to Hinduism’. As far as the religious sphere, most interviewees in Italy expressed a higher level of satisfaction compared to interviewees in Spain. In Italy, the Sikh population is currently trying to register Sikhism as a registered religion and organising itself to have a presence in the local politics.

Indians mostly do not take part in the local festivals, mainly because of their very little knowledge about the host festivals. But they do celebrate Indian festivals in their religious temples or by renting public buildings. According to interviewees, all religious groups invite the hosts to take part in their religious events (Nagar Kirtans, Poojas and Yoga camps), but the response of the local community is very limited. Kashmir, 57, male, Gurudwara head from Barcelona, states that ‘We celebrate our Sikh festivals in Gurudwara sahib and invite local people to take part in our festivals, but only few people from the native population come to join us’.

In the cultural preference, change in immigrants’ preferences for food, music and art under the effect of the host culture and the impact of immigrants on the arts, cuisine, music and fashion of the host society is included. In Spain and Italy, we still do not find fusion of Indian music and cuisine with the host ones, like we see in other countries of Indian diaspora (e.g. the UK Bhangra Pop music and Manchester’s Curry lane). Some of the Indians stick to their traditional Indian vegetarian diet mainly because of their religious beliefs, like Gagandeep, 21, male, a baptised Sikh from Hospitalet de Llobregat, says ‘I have my vegetarian diet. I don’t eat Spanish food because they put meat or eggs in all dishes’. But most of Indians have added the local dishes in their daily meals, like Gaurav, 24, male, a salesman in Madrid, Spain, says ‘I love Mediterranean food. My favourite food is Pizza and Pasta; occasionally I eat Indian food also, but now I find it very spicy’. An important change is occurring in some supermarkets in major cities of both countries: now, they provide a separate section for South Asian food, in order to satisfy the needs of the immigrant population. Moreover, Indian Bollywood music and dance is also becoming popular in some parts of Spain. In the future, it will be interesting to see when and how the Indian cuisine and music get fused with those of the host countries, and hybrid music and cuisine come out from this fusion.

For the Indian immigrants, the host language remains one of the main integration problems in both countries. Majority of Indians have none to very little knowledge of host languages, which affects their personal, social and economic life, and level of integration into the host society. Even within the Indian community, a majority of Sikhs have relatively little language skills as compared to Indian Christians and Hindus. Sometimes, the skills learned in India become useless because of the lack of host languages and the long waiting time for the homologation of professional degrees in both countries, like Nitu, 30, female, a housewife in Roma, explains that ‘for me language was the first main problem. In India, I was a nurse in a hospital, but here [Italy] without Italian, I failed to get any job. Even my nursing degree is not homologised here, so all skills learned in India becomes useless here, that’s why I don’t want to settle here permanently’. Indian immigrants who are engaged in sales and health care services (mostly Sindhi shopkeepers or Malayali nurses) learn language quickly to fulfil the requirements of their jobs; conversely, for the agricultural labour and other manual factory or construction workers (mostly Punjabis), the language learning process is very slow. As Pritpal, 47, male, a construction worker in Barcelona, states that ‘I am living in Spain for the last 13 years, but still I speak only functional Spanish. Mostly I work with Indians, so I don’t feel any need to learn it’.

Indian families take huge interest in the language learning of their children. Most of the Indian children speak their native language (Punjabi, Hindi, Sindhi or Malayalam) or English with their parents and the host country’s language (Spanish or Italian) with their siblings and friends. Generally, parents are worried about the lack of English in public schools and preservation of their native languages. Gopal, 47, male, a shopkeeper in Santa Cruz de Tenerife explains: ‘With my children I converse in Sindhi, and sometimes English, so that they can learn their mother tongue, and a foreign language. The Spanish they will learn in schools, but mother tongue is necessary for their attachment to their own culture and English is compulsory for their future. With English they can migrate and work at any place’. While retention of migrants’ own language may offer no advantage in educational attainment, it may nevertheless do so in relation to the migrant’s sense of belonging and access to ethnic networks. Recently, in many Gurudwaras and Hindu temples, language of origin courses has been organised for children; Gurmukh, 63, male, head of Gurudwara committee in Barcelona, explains ‘In Gurudwara we organise summer camps for children, where we teach language and religion. It is very important otherwise they will forget their mother tongue’.

### Identificational integration

The sense of belonging is a fundamental sign of integration, as already stressed. It shows the level of attachment and the loyalty with the host communities. The Indian immigrants have shown very little attachment to the host countries, as they believe that their true home is in India, and Italy and Spain are only their temporary shelters. As in the words of Balwinder, 48, male, a factory worker living in Barcelona for the last 21 years, ‘my true home is in India. I have lived almost half of my life here [in Spain], but still my feelings are attached to the places where I have spent my childhood and teenage’. Generally, the Indian community has not experienced any racial or ethnic discrimination in both countries. They have very positive thoughts about the host community, but some explain about a shift in the host’s attitude towards immigrants because of the increasing incidences of crime committed by immigrants, like in the words of Kamal, 30, female, a shopkeeper in Barcelona, ‘Generally, native people are very good. They treat all immigrants very well, but due to the increasing crime mainly because of Romanians, Moroccans or African immigrants, now they feel afraid of immigrants and don’t want more immigration’.

In the Indian community, the concept of identity is multi-layered. Most of the Indians first identify themselves with their place of origin in India (like Punjabis, Haryanvi, Keralite or Sindhis), then with their religion (Sikh, Hindu, Muslim or Christian), then with their national status (Indians or foreign citizens) and lastly with their new place of residence (Italian or Spanish). Mostly they feel that in the minds of the host community, their identity will always be an immigrant. Gurpreet, 30, male, a salesman from Madrid, explains his identity as ‘I am a Punjabi Sikh, from India, this is my identity. When I will get the Spanish nationality, I will add another layer of being Spanish to it. But I will remain Indian forever -an immigrant- in the eyes of local people’. A very crucial aspect of immigrant integration is the degree to which the host society permits insertion of immigrants into the society through its policies, programs and integration initiatives (Sardinha 2009). While explaining the attitude of Italian people regarding the immigrants, Ranjit, 48, male, an agriculture worker from Latina, says ‘Italian people don’t want to make us ‘Italian’, they need us for work. We are welcome here until we serve their purpose, when we start asking for our rights, they will label us as foreigners and through us out’.

Many Indians (especially Sikhs), complain about their confused identities in both countries, as the hosts often confuse them with Pakistanis or Afghanis in Barcelona and Bengalis in Rome and Madrid. It is basically owing to the large concentration of other immigrant groups in these regions. Baptised Sikh males are confused with Muslims and owing to this confusion sometimes, they receive harsh treatment from hosts. Harjeet, 28, male, a baptised Sikh from Barcelona, explains ‘often local people confuse me with Pakistanis and sometimes they call me Bin Laden or terrorist. Sometimes I feel hurt, but I know they are ignorant and don’t know anything about me and my religion’. Among immigrants, the emergence of hyphenated identities is a symbol of integration and the acceptance of host society as their new home. Some Indians in both countries have started presenting themselves as Spanish-Sindhis or Italian-Sikhs.

## Integration and transnationalism: quite conflicting processes

As well acknowledged, transnationalism and integration are two interconnected processes and they are not necessarily mutually exclusive (Cassarino 2004; Carling and Vatne 2014). However, their outcomes are not always easy to schematise: integration tends to strengthen the linkages with the country of residence, while transnationalism reinforces those with the country of origin. According to transnationalism, frequent movements between host and home countries and exchange of information, which have been intensified through the advancement of technologies, can increase migrants’ opportunities to remain connected with the country of origin. However, transnational migrants may decide to return to their homeland to invest with the resources acquired from the host country, regardless of the level of integration abroad (De Haas and Tineke 2010). From another perspective but similarly, according to the social network theory, migrants are likely to move and eventually to return home if they maintain ties with their former place of settlement (Glick Schiller 1999).

**Fig. 3:**
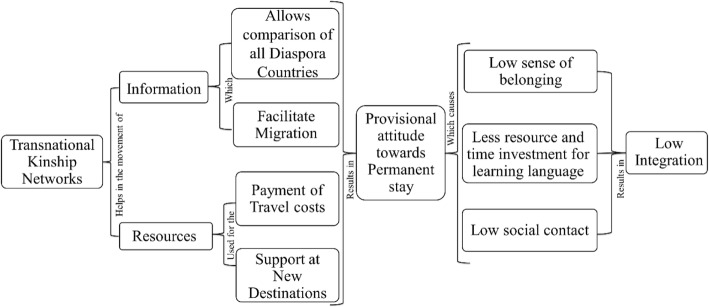
The effects of transnational links of Indian immigrants in Spain and Italy on their level of integration into the host societies. Source: compiled by the first author on the basis of in-depth interviews during 2016–2017

Transnational networks provide a mechanism for the flow of information and resources in the global Indian diaspora, which help them to move from one country to another. It often results in the provisional attitude towards their permanent stay in the first host country, where the conditions are not satisfactory. In particular, when they compare their standard of living with that of their relatives or friends in other destinations countries, it discourages them to nurture a sense of belonging and attachment to the country of residence and in putting effort to increase their degree of integration into the host society (Fig. 3). In particular, the information they receive from their transnational community, about work and living conditions elsewhere, facilitate their plan to move on to other countries, where they think they can find better jobs and welfare conditions for themselves and their children.

As explained by Gurjeet, 42, male, a restaurant worker in Barcelona, ‘at present the flow of information and transport is very fast. People receive information from their relatives and friend, and compare the opportunities at different places. If they find that there are more opportunities at other destination, they move on there’. We believe that this mechanism may encourage the so-called instrumental use of citizenship that the 2008 economic crisis has contributed to increase (Finotelli et al. 2017) and according to which the citizenship of the host country is used by immigrants to move to EU or non-EU destinations. Sandeep, 32, female, a housewife from Brescia, illustrates her plan ‘my sister lives in the UK. She told me that life in the UK is very good. There are many jobs for women also, and for kids the best English education is available at relatively cheap price. So, when we get red passports [citizenship], we will move on there’. In Spain, 22 out of 48 and, in Italy, 24 out of 38 respondents have declared the intention to re-emigrate after having received the citizenship of the host country. Moreover, some of them affirm that their friends or relatives have already moved to the UK or to Canada, after having obtained the host nationality. In Italy, the number of people interested in onward migration is higher than Spain. Nevertheless, our findings do not allow us to conclude that Indians are making an instrumental use of citizenship, even though they point to a possible instrumental approach to citizenship acquisition: obtaining the citizenship of the country of residence also in order to move to other destinations, especially when the integration process and thus the sense of attachment to the host country appears fragmented and the transnational links within the diaspora quite strong, as in the case of the Indian community in both countries (Prieto et al. 2018).

Finally, the perceived provisional stay in Spain and Italy, together with the objective difficulties met in learning a language, which is quite different from their own languages, discourages Indians to put the necessary efforts in learning the language and culture of the host countries. On this regard, Sarabjot, 38, male, a serviceman in Barcelona, says ‘I don’t want to learn Spanish or Catalan perfectly because I am not going to live here forever. I have relatives in the UK. They are going to sponsor my visit there. If plan goes well, I will settle there permanently’.

## Conclusions

Integration can be understood as ‘processes that increase the opportunities of immigrants and their descendants to obtain the valued ‘stuff’ of a society, as well as social acceptance, through participation in major institutions such as the educational and political system and the labour and housing markets’ (Alba and Foner 2016: 5). From the discourse of our interviewees, we found that Indian immigrants in Italy and Spain have limited access to the valued ‘stuff’ of the host societies and lacked the acceptance from the host societies (Penninx and Garcés-Mascareñas 2016: 14). They have very low integration into different spheres (defined by Heckmann et al. 2001) of the host societies. At present, the process of integration is working properly only for few segments of the Indian community in both countries. The inherent diversity of Indian immigrant population, in terms of their gender, level of education, employment, religion and legal status, which our qualitative work has highlighted, affects the pace of the integration into the host society. As well acknowledged, more educated, highly skilled and legally resident immigrants integrate faster and successfully in the host society (Busetta 2016). The majority Sikh population, who is mainly irregular, low educated, and low skilled, is less integrated as compared to other religious groups of Indian origins (i.e. Hindus and Christians) in both countries. Moreover, the small segment of Indian community, which is integrating to the host society, is also mainly limited to the economic sphere. It results in what we have called the ‘fragmented integration’, which is mainly caused by the uncertain attitude of Indians towards permanent stay in both countries.

As discussed, the discrimination faced by Indian immigrants in the labour market, housing, education institutes and the civil society, especially because of their language, religion, and physical aspects, negatively affects their level of integration into the host societies (also see Busetta et al. 2018). Similarly, the lengthy regularisation and naturalisation processes, degraded public education system and lengthy process of homologation of professional degrees adversely affect their perceptions and satisfaction from the host countries and further discourage the efforts required for the building of a strong sense of attachment and belonging to the host countries. Additionally, it encourages them to move on to other countries, such as the USA, Canada or the UK. The English education for their children, better job opportunities, kinship networks, high social capital and a better value attached to these countries in the diaspora are among the major pull factors that attract Indian immigrants to these countries. On this regard, most of our interviewees are waiting for the European passports to migrate to their desired destinations. The transnational networks provide them necessary information, resources and assistance for immigration to these countries, by reducing the costs. This possibility to get settled in another more developed country decreases their interest in a successful integration process and a permanent settlement in Italy and Spain.

On the part of the host governments, firstly, investment in the host language learning programs can make a significant contribution in improving the pace of integration of Indian immigrants. Host languages which are now acting as barrier can become a bridge, by facilitating the exchange of feelings and ideas between Indian immigrants and host societies. Secondly, the vocational training programs for women can help them to enter in the labour market, which will improve their level of economic integration into the host labour market. Thirdly, the simplification of regularisation laws can improve the upward legal mobility of irregular Indian immigrants, which will improve their chances to get permanently settled in the host countries. Fourthly, the registration and official recognition of their religions can facilitate the sense of belonging among them. And finally, the strict control over discrimination of all types can encourage the different sections of the Indian society to integrate with the main stream host societies.

While comparing the level of integration of Indian immigrants in both countries, some differences emerged. The most important one pertains to the recognition of rights of irregular immigrants. In Spain, the right to be registered in municipal office; the right of education and enrolment in language courses; the right to use public health facilities; the right to use social services, libraries and community centres; and finally the *Arraigo* law helped the most part of Indians to improve their social and economic status. On the contrary, the almost total denial and neglect of irregular immigrants in Italy (with the exception of urgent medical cares and children’s education, as regulated by the *Turco-Napolitano law* in 1999) has contributed to the isolation and the marginalisation of Indian immigrants. Time is the most relevant factor for the integration process for both immigrants and receiving societies. For first-generation immigrants, integration needs a second socialisation that requires many intellectual and emotional costs. However, second-generation immigrants are expected to pass through possibly demanding forms of bi-cultural socialisation and identity formation. In future, it will be interesting to see how the second generation of Indian immigrants in both countries evolve their relation with the host societies. Lastly, we want to acknowledge that, although they contribute to fill an important gap in migration and integration studies, all the empirical findings reported in this study are based on the perceived and self-reported experience of Indian immigrants in Italy and Spain; therefore, they are not necessarily representing the whole Indian community, which is a very heterogeneous and multifaceted immigrant group, and for this reason deserves further research.

## Footnotes

1 The continuous regularisation with *Arraigo* (rootedness) was introduced in Spain through Immigration Act 4/2000. With its two modalities (*laboral and social*), *Arraigo* works as a permanent regularisation mechanism. Through this legal apparatus, immigrants are given the possibility of applying for regularisation after two (*Laboral Arraigo*) or three (*Social Arraigo*) years of registration in the municipality, providing a clean criminal record, an employment contract and a ‘social insertion’ certificate, provided by the town council of residence.
